# Editorial: Protozoal infections: Treatment and challenges

**DOI:** 10.3389/fcimb.2022.1002602

**Published:** 2022-08-31

**Authors:** Vipan Kumar, Gaurav Bhargava

**Affiliations:** ^1^ Department of Chemistry, Guru Nanak Dev University, Amritsar, India; ^2^ Department of Chemical Sciences, Inder Kumar Gujral (IKG) Punjab Technical University, Kapurthala, India

**Keywords:** neglected tropical diseases, malaria, anti-plasmodial, pyrimidine biosynthesis, kinase inhibitors, Anti-leishmanial

Protozoal infections caused by *Plasmodium falciparum*, *Trypanosoma cruzi*, *Leishmania mexicana*, *Giardia lamblia*, and *Trichomonas vaginalis*, among others, are commonly referred to as Neglected Tropical Diseases (NTDs) because they receive little attention in terms of surveillance, prevention, and treatment. These group of infectious diseases are prevalent in 149 countries with tropical and subtropical environments and are closely linked with poverty. More than 1.5 billion people worldwide are afflicted by at least one NTD, with populations that lack access to sufficient sanitization, clean water, and healthcare as well as those who live close to infectious disease carriers being the most at risk. The tenth anniversary of the London Declaration on Neglected Tropical Diseases (NTDs) in 2022 marks a watershed moment in which governments, pharmaceutical companies, foundations, and non-governmental organisations (NGOs) pledged to work together to eradicate NTDs. The pandemic of COVID-19 has been a historic setback for the NTD agenda. It has harmed neglected populations disproportionately, not only increasing global poverty but also having a direct impact on NTD interventions. COVID-19 has frequently and severely impacted NTD services, causing disruptions in 44% of countries, according to a WHO survey. The papers published in this Research Topic are primarily concerned with alternative approaches for NTDs, particularly malaria and leishmaniasis (Special topic “Protozoal infections: Treatment and challenges).

Jahnmatz et al. assessed cross-sectional pre-existing memory B-cells (MBCs) and antibody responses against six well-known *P. falciparum* antigens and measured their associations with previous infections and time to clinical malaria in Kenyan children. Children who exhibited three or more antigen-specific MBC or antibody responses at baseline had a reduced probability of contracting malaria during the next *P. falciparum* transmission season. They come to the conclusion that malaria protection is related with a wider range of merozoite antigen-specific MBC and antibody responses. According to Carvalhoet al., boromycin has a highly effective anti-plasmodial effect against both the zoonotic *P. knowlesi* and *P. falciparum*. Unlike tetracyclines, boromycin quickly killed asexual stages of both Plasmodium species at low concentrations (1 nM), including strains of *P. falciparum* that were multidrug resistant (Dd2, K1, 7G8). Additionally, low nanomolar concentrations of boromycin were effective against *P. falciparum* stage V gametocytes (IC50: 8.5 ± 3.6 nM). The apicoplast was excluded as the primary target based on a mechanism of action investigation. Although significant ionophoric activity in potassium channels was observed, it was insufficient to fully account for the compound’s antiplasmodial activities.

Malaria resistance has also been linked to pyruvate kinase deficiency in both population studies and experimental models. The diminution of ATP and the elevation of 2,3-biphosphoglycerate (2,3-BPG) concentration are two of the main pyruvate kinase deficient-cell disorders. Morais et al. investigated the effect of a synthetic form, 2,3-DPG, on the intraerythrocytic developmental cycle of *P. falciparum in vitro*. The findings demonstrated that 2,3-DPG exposure adversely affected parasite development, which in turn had an impact on parasite maturation. The pyrimidine biosynthesis pathway is critical for cell growth and proliferation. Since the malarial parasite lacks a functional pyrimidine salvage pathway, RNA and DNA synthesis is entirely dependent on *de novo* synthesis. The activity of the parasite transcarbamoylase has been identified as at least one of the targets of Torin2, a powerful but non-selective antimalarial. Wang et al. focused on an allosteric pocket that supports the catalytic pathways to provide a summary of the research on the *P. falciparum* ATCase structure. They performed a fragment-based screening to find hits using the crystal structures of the malarial aspartate transcarbamoylase.

Sussman et al. described the presence of phylloquinone (PK/vitamin K1) in *P. falciparum* and talked about its possible origin. Exogenous PK attenuated the effects of atovaquone on parasitic growth and respiration, indicating that this metabolite can be transported from extracellular environment and that the mitochondrial electron transport system (ETS) of *P. falciparum* is capable of interacting with PK. This study emphasises the importance of PK in plasmodial metabolism, which will require further investigation in order to identify new antimalarial drug targets. Wilson and colleagues used the FAF-Drugs4 server to test 885 AfroDB-retrieved compounds, resulting in the discovery of 91 ADMET-acceptable compounds. ZINC13374323 and ZINC13365918 were chosen as potential lead compounds. ZINC13374323, also known as aurantiamide acetate, is a component of *A. annua* that acts as an inhibitor of *P. falciparum thymidylate monophosphate kinase* (*Pf*TMPK). The complex of *Pf*TMPK and ZINC13374323 has similar RMSD and RMSF to that of the protein in association with its native substrate, TMP, according to molecular dynamics simulations. *In vitro* testing revealed that aurantiamide acetate’s IC_50_ for anti-plasmodial activity was 69.33 μM.

Sakyi et al. used homology modelling to establish the structural model of *Leishmania donovani* 24-sterol methyltransferase (LdSMT) in order to find possible 24-SMT inhibitors through virtual screening, scaffold hopping, and *de-novo* fragment-based design. Six potential novel inhibitors were identified, with binding energies similar to 22,26-azasterol, the main inhibitor of LdSMT (7.6 kcal/mol). The binding mechanism determined that Tyr92 was necessary for binding, and estimates of the Poisson-Boltzmann surface area (MM-PBSA) and molecular dynamics simulations confirmed this. The review by Santos and Rebello mainly focused on the development of drug repositioning as a practical technique for the treatment of mucosal parasites while evaluating potential candidates that target protozoans which infect mucosal surfaces. When taken as a whole, these studies emphasise the significance of developing alternative strategies and drug repositioning for combating NTDs.

## Author contributions

The authors listed have made a substantial, direct, and intellectual contribution to the work and approved it for publication.

## Acknowledgments

All authors who contributed to the Research Topic “Protozoal Infections: Treatment and Challenges” are gratefully acknowledged by the editors. Each reviewer who has contributed and whose invaluable assistance is essential to the journal’s success is acknowledged separately.

## Conflict of interest

The authors declare that the research was conducted in the absence of any commercial or financial relationships that could be construed as a potential conflict of interest.

## Publisher’s note

All claims expressed in this article are solely those of the authors and do not necessarily represent those of their affiliated organizations, or those of the publisher, the editors and the reviewers. Any product that may be evaluated in this article, or claim that may be made by its manufacturer, is not guaranteed or endorsed by the publisher.
